# The effect of web based depression interventions on self reported help seeking: randomised controlled trial [ISRCTN77824516]

**DOI:** 10.1186/1471-244X-6-13

**Published:** 2006-04-05

**Authors:** Helen Christensen, Liana S Leach, Lisa Barney, Andrew J Mackinnon, Kathy M Griffiths

**Affiliations:** 1 Centre for Mental Health Research, The Australian National University, Canberra ACT, Australia

## Abstract

**Background:**

To date, there has been very little work investigating behaviour changes induced by interventions that are designed to increase help seeking. The present paper examines the effects of two Internet depression websites on help seeking.

**Methods:**

414 individuals with elevated scores on a depression assessment scale were randomly allocated to a depression information website, a cognitive-behavioural skills training website (CBT) or an attention control condition. Reports of help seeking for specific treatments, from specific sources and for categories of treatments were assessed.

**Results:**

Relative to the control, the depression information site was associated with decreases in seeking support from friends and family, the use of music and of everyday treatments and no increase in seeking evidence based interventions. The CBT site was associated with the report of help seeking for CBT, massage and exercise.

**Conclusion:**

Methods to encourage the use of evidence-based treatments need further research to determine whether the assistance sought is evidence based and whether there are unintended effects.

## Background

There is recognition from the results of representative epidemiological surveys that most adults with mental health problems do not receive treatment [1,2].

Despite this, there has been very little research investigating the effect of interventions designed to increase help seeking. Although the effects of educational pamphlets [3], voluntary screening [4] and educational programs in schools [5] on help seeking have been reported, these studies typically neither used randomised controlled designs nor investigated the types of treatment sought. One randomised controlled study [6,7] did examine the effect on help seeking attitudes of a consumer guide to depression. This study provided information about a range of treatments with demonstrated support for efficacy based on a systematic review of the evidence [8]. A major weakness of this study was that the short brochure used in the control condition also provided information about evidence based treatments for depression.

Using a randomised controlled trial design in which the efficacy of the active intervention arms had been established [9], the current study examined the effect of a depression information website (BluePages) on self reported help seeking. The effects were compared to those produced by a cognitive-behavioural skills training website (CBT) (MoodGYM) and an attention control condition. The inclusion of results from the MoodGYM arm of this trial constitutes an additional active comparison group. MoodGYM has been shown to be of equal effectiveness to BluePages in reducing depression but it does not directly encourage participants to seek additional help.

## Methods

### Participants

A screening questionnaire was posted to 27,000 individuals aged 18 to 52 randomly selected from the Canberra electoral roll. 6130 people (22.7% response rate; 24.6% excluding those returned to sender) returned the screening questionnaire and 657 respondents met inclusion criteria. The screening questionnaire included a brief questionnaire (about two printed pages) assessing basic demographic information, education level, depression symptoms, help-seeking and internet use. Respondents were not eligible for inclusion in the trial if they; (i) scored below 12 on the Kessler Psychological Distress Scale (K10)[10], (ii) indicated they did not have Internet access at home or at work, (iii) indicated they did not wish to participate in an intervention, or (iv) were under psychiatric or psychological care. Of those who responded, 525 participants met these criteria, returned a letter of consent, and were randomised to the interventions. 414 participants provided post intervention depression scores and these individuals formed the sample analyzed here. The sample comprised 114 men and 300 women. The mean respondent age was 36.8 years (SD = 9.3), and the mean Kessler score was 17.5 (SD = 5.0) at screening.

### Design

The present study used data from a recently completed randomised controlled trial of two Internet sites [9,11]. One of these Internet sites, BluePages [12], delivers information about a range of psychological, medical and alternative treatments and recommends those supported by scientific evidence. At the time of the trial, the BluePages website reviewed 5 medical, 7 psychological and 36 alternative treatments, recommending 3, 5 and 13 of these, respectively. The recommended therapies were based on systematic reviews of treatments [8,13]. The second site, MoodGYM, provides online cognitive behaviour therapy (CBT). The site consists of five modules that provide cognitive behaviour therapy, strategies to manage emotional distress, relaxation and problem solving. The site provides downloadable summary pages at the end of each module, which provide individualized symptom scores and homework completion outcomes. The site offers a user profile which tracks changes in anxiety, depression and dysfunctional thinking across modules. A control condition involved telephone contact with an interviewer but no access to the websites. Participants were randomly assigned to receive either; a) BluePages (n = 165, at endpoint n = 136), b) MoodGYM (n = 182; at endpoint n = 121) or c) a control condition (n = 178, at endpoint n = 157) in which an interviewer asked open-ended questions about factors that may influence depression. Participants were randomised to interventions using the SPSS SELECT CASES random selection option. A flow chart of participation in the trial is included in a previous publication [9].

### Procedures

Participants completed questionnaires delivered through the mail at screening, pre-test, endpoint and at six months after the trial. Those in the BluePages and MoodGYM conditions were provided with a login identification number and a manual containing information about MoodGYM or BluePages. This manual outlined which sections or modules of the sites participants were to complete for each of 5 weeks. Interviewers maintained weekly telephone contact with participants in all conditions over the period of the intervention (a total of 6 contacts of approximately 10 minutes each).

Interviewers were provided with separate instruction booklets for each participant containing verbatim instructions for each of the weekly contacts. Participants randomised to the control intervention were phoned weekly for 10 minutes to discuss lifestyle and environmental factors that may influence depression. A different topic was discussed each week; 1) physical and artistic activities, 2) education and hobbies, 3) social financial and family roles, 4) work habits and stress, 5) physical health, medication and pain, and 6) nutrition and alcohol. At the end of the intervention period and at six months all participants were sent a questionnaire. The study was approved by the Human Ethics Committee of the Australian National University. Participants provided written informed consent.

### Measures

#### Demographic

Age, sex, marital status, years of education and previous history of depression were assessed.

#### Depression

Severity of depression at pre and post intervention was assessed using the 20 item Centre for Epidemiological Studies Depression Scale (CES-D) scale (range 0 to 60) [14]. Higher scores represent greater psychological distress with scores 16 or higher indicating clinical depression [14]. To compare to Jorm et al., [6] CES-D depression scores at pre-intervention were then grouped into five intervals 'Low' (0–9), 'Mild' (10–19), 'Moderate' (20–29), 'High' (30–39) and 'Severe' (40–60).

#### Help-seeking

(i) *Treatments sought by participants*. At post intervention and at six months respondents were asked 'We would now like you to tell us which of the following treatments or activities (if any) you have used in the past 2 months to cope with depression.' Respondents were then given a list of items and answered either 'yes' or 'no' to each item. This list of self-help and standard professional therapies was compiled from a systematic review of treatments for depression [8,13]. Only items with more than 10% endorsement rate were analysed. The list of these items contained nine evidence-based treatments: one medical professional treatment (use of anti-depressant medication), two psychological treatments (CBT and self-help books) and six alternative treatments (exercise, yoga, massage, relaxation, cut out alcohol, use of vitamins); and eight non-evidence based treatments: one psychological treatment (counseling), and seven alternative treatments (eating chocolate, music, being with pets, doing enjoyable things, meditation, avoiding caffeine, use of alcohol).

(ii) *Seeking help from particular sour*ces was also assessed. Participants were asked whether they had sought help from a 'general practitioner', 'counsellor or psychologist' or from 'friends or family'.

(iii) *Help seeking treatments by category *using the categorization introduced by Jorm et al [6] were also assessed. The treatments were grouped into four categories: Everyday treatments included family and friends, exercise, eating chocolate, listening to music, being with pets and doing more things you enjoy. Everyday actions were so named because they reflected activities which are not necessarily seen to be therapeutic but which are frequently undertaken or experienced in daily life. Complementary treatments included yoga, massage, relaxation therapy and meditation. Complementary treatments were those identified through a systematic search of the literature, but which were not commonly identified in clinical practice guidelines. Dietary treatments included avoiding caffeine and drinking alcohol. Professional treatments included seeking the help of general practitioners, counselors and psychologists, anti-depressants, cognitive-behavioural therapy, counseling and reading self-help books. A previously used category – non-prescription treatments (which includes painkillers, St John's wort, fish oils, vitamins and 'cut down on alcohol') – was not able to be used in the present analysis because most items were too infrequently endorsed. Two components within this category, 'vitamins' and 'cut out alcohol'- were included in the dietary category. Thus the everyday category had six treatments, the complementary category four treatments, the dietary category four treatments and the professional category six treatments. These categories were originally derived from a principal components analysis of a range of treatments found in the literature [6].

### Statistical analysis

The first set of analyses addressed the question of whether the interventions were associated with reports of help seeking. It consisted of a series of separate logistic regressions assessing the impact of BluePages compared to the control condition, and BluePages compared to the MoodGYM site on help seeking for each of the specific treatments used (e.g., antidepressant medication). A second analysis assessed the effect of these the two interventions and the control condition on help seeking from different sources of help (professionals/family). A further set of four linear regressions examined whether those in the BluePages intervention compared to the two other interventions used different categories of treatments.

BluePages was selected as the comparison condition in preference to the control condition because we hypothesized it would lead to greater help seeking for evidence-based treatments than the other two conditions. Supplementary analyses addressed the question of whether the reported seeking of help from a specific treatment or source was associated with a reduction in depressive symptoms. Separate linear regressions were used to examine the reported use of each specific treatment and its association with depression (CES-D scores) at post intervention and at six months. A further four linear regressions examined whether the category types of treatment predicted depression. A second set of supplementary analyses investigated the relationship between the severity of depression and the reported uptake of specific treatments or sources, controlling for intervention type. A series of separate logistic regressions were used to assess the effect of the severity of depression (CES-D scores) at pre-intervention on the use of each treatment. Four linear regressions were used to examine the impact of depression on the number of every day, complementary, dietary and professional treatments used. No formal adjustment was undertaken for the number of comparisons that were tested. However, the majority of effects reported would remain significant even if correct using a extremely conservative approach such as the Bonferroni method. Nevertheless, the possibility of inflated Type I error arising from evaluating multiple treatments should be borne in mind.

## Results

### Demographic characteristics

There were no significant differences in age, sex and CES-D score between the intervention conditions at baseline [9,11]. Participants who completed the post-intervention questionnaire did not differ in age, sex or years of education or the CES-D at endpoint in comparison to those who did not return the questionnaire, although the dropouts had higher depression scores at baseline (F (1,523) = 5.15, p = 0.24).

### Effects of the sites on depression symptoms

Both BluePages and MoodGYM significantly reduced depression symptoms at post intervention relative to the control condition. Scores reduced 4.5 points (1.8 to 7.3) (95% confidence interval) for MoodGYM and 3.6 points (1.0 to 6.3) for BluePages relative to the control condition.

### Effects of the interventions on help seeking

Frequencies for the reported use of each treatment are shown in Table 1. The results for the logistic and linear regressions are also displayed. Compared to BluePages, MoodGYM participants were more likely to report using cognitive behavioural therapy, exercise and massage. Both MoodGYM and control participants were more likely to report listening to music than were BluePages participants. Compared to BluePages, the control group was more likely to report seeking the help of family and friends. Both MoodGYM and the control condition were associated with the reported use of more everyday treatments. MoodGYM was also associated with the seeking of more complementary and professional treatments. These analyses were repeated as six months (Table 2). A number of the differences between the intervention groups were no longer significant. However, MoodGYM participants were still more likely to say they had used cognitive-behavioural therapy than those in BluePages (OR = 2.73; p = 0.011), and to use everyday treatments (B = .48, p = .044). After six months those in the control group were still more likely to have listened to music than those in the BluePages intervention (OR = 2.10, p = .005), and were now more likely to report the use of pets as a form of treatment than those in BluePages (OR = 1.86, p = .018). Both MoodGYM participants and the control group were more likely to use alcohol as a form of treatment at six months, than those in BluePages (OR = 2.26, p = .031, OR = 2.13, p = .037).

**Table 1:** Treatment use percentages for each condition and summary of regressions predicting treatment use from intervention type

	BluePages	Control	MoodGym	Control – BluePages			MoodGym – BluePages		
Specific Treatments	(N = 136)	(N = 157)	(N = 121)	OR	p	95%CI	OR	p	95%CI

*Evidence based*									
Antidepressants	27.9	25.5	24.8	.88	.634	.53 – 1.48	.85	.568	.49 – 1.48
CBT	11.0	7.6	28.9	.67	.320	.30 – 1.48	3.28	.000**	1.69 – 6.38
Self-help books	23.5	19.7	27.3	.80	.432	.46 – 1.40	1.22	.491	.69 – 2.14
Exercise	56.6	63.7	68.6	1.34	.217	.84 – 2.15	1.67	.049*	1.01 – 2.79
Yoga	9.6	9.6	13.2	1.00	.999	.46 – 2.18	1.44	.356	.66 – 3.14
Massage	13.2	15.9	24.0	1.24	.517	.65 – 2.39	2.07	.028*	1.08 – 3.95
Relaxation therapy	12.5	7.6	18.2	.58	.169	.27 – 1.26	1.57	.207	.78 – 3.09
Cut out alcohol	14.0	19.0	19.0	1.22	.540	.64 – 2.32	1.45	.277	.74 – 2.81
Use vitamins	10.3	15.9	14.9	1.65	.160	.82 – 3.32	1.52	.269	.72 – 3.21
									
*Non-evidence based*									
Counselling	8.1	14.0	11.6	1.85	.114	.86 – 30.97	1.49	.349	.65 – 30.14
Eating chocolate	19.9	22.9	27.3	1.20	.523	.69 – 2.11	1.51	.162	.85 – 2.71
Listening to music	42.6	59.9	56.2	2.01	.003**	1.26 – 3.20	1.73	.031*	1.05 – 2.83
Being with pets	41.9	52.9	50.4	1.56	.062	.98 – 2.47	1.41	.173	.86 – 2.31
Doing things you enjoy	57.4	60.5	59.5	1.14	.584	.71 – 1.82	1.09	.727	.67 – 1.80
Meditation	13.2	12.7	19.0	.96	.900	.48–1.89	1.54	.209	.79–3.01
Avoiding caffeine	11.0	10.2	11.6	.92	.816	.44–1.93	1.06	.891	.49–2.29
Use alcohol	18.4	23.6	19.0	1.37	.280	.78-.242	1.04	.898	.56–1.95
									
Sources of Help									
GP	22.1	22.3	28.1	1.01	.962	.58 – 1.76	1.38	.265	.78 – 2.43
Counsellor/psychologist	10.3	15.9	18.2	1.65	.160	.82 – 3.32	1.93	.072	.94 – 3.98
Family and friends	56.6	71.3	64.5	1.91	.009**	1.18 – 3.10	1.39	.200	.84 – 2.30

Categories of Treatments	Mean (SD)	Mean (SD)	Mean (SD)	B	p	95% CI	B	p	95% CI

									
Professional treatments (Scale: 0–6)	1.03 (1.25)	1.05 (1.37)	1.39 (1.47)	.16	.893	-.29 – .43	.36	.036*	.02 – .69
Everyday treatments (Scale: 0–4)	2.75 (1.65)	3.31 (1.68)	3.26 (1.60)	.19	.004**	.18 – .94	.51	.013*	.11 – .92
Complementary treatments (Scale: 0–4)	0.49 (0.81)	0.46 (0.77)	0.74 (0.94)	.10	.786	-.22 – .17	.26	.014*	.05 – .46
Dietary treatments (Scale: 0–6)	0.54 (0.78)	0.66 (0.83)	0.64 (0.89)	.10	.198	-.07 – .32	.11	.360	-.10 – .31

Notes: Professional treatments included; GP, Counsellors and psychologists, anti-depressants, cognitive-behavioural therapy, counselling and reading self-help books. Everyday treatments included; family and friends, exercise, eating chocolate, listening to music, being with pets and doing more things you enjoy. Complimentary treatments included; yoga, massage, relaxation therapy and meditation. Dietary treatments included; cutting out alcohol, taking vitamins, avoiding caffeine and drinking alcohol.

**Table 2:** Treatment use percentages at six month follow-up for each condition and summary of regressions predicting treatment use from intervention type

	BluePages	Control	MoodGym	Control – BluePages			MoodGym – BluePages		
Specific Treatments	(N = 114)	(N = 130)	(N = 102)	OR	p	95%CI	OR	p	95%CI

*Evidence based*									
Antidepressants	25.4	26.2	28.4	1.04	.899	.58–1.85	1.16	.620	.64–2.13
CBT	9.6	6.9	22.5	.70	.441	.28–1.75	2.73	.011*	1.26–5.92
Self-help books	24.6	22.3	15.7	.88	.678	.49–1.60	.57	.108	.29–1.13
Exercise	54.4	56.9	64.7	1.11	.691	.67–1.84	1.54	.124	.90–2.66
Yoga	13.2	10.8	8.8	.80	.566	.37–1.73	.64	.314	.27–1.53
Massage	16.7	15.4	18.6	.91	.785	.46–1.80	1.15	.706	.57–2.31
Relaxation therapy	12.3	6.2	10.8	.47	.102	.19–1.16	.86	.732	.37–2.00
Cut out alcohol	13.2	15.4	15.7	1.20	.621	.58–2.47	1.23	.597	.57–2.63
Use vitamins	11.4	15.4	12.7	1.41	.366	.67–2.99	1.14	.762	.50–2.58
									
*Non-evidence based*									
Counselling	11.4	12.3	11.8	1.09	.828	.50–2.38	1.04	.934	.45–2.39
Eating chocolate	21.1	23.8	30.4	1.17	.60	.64–2.15	1.64	.117	.88–3.04
Listening to music	47.4	65.4	53.9	2.10	.005**	1.25–3.51	1.300	.337	.76–2.22
Being with pets	38.6	53.8	45.1	1.86	.018*	1.11–3.09	1.31	.334	.76–2.25
Doing things you enjoy	56.1	55.4	67.6	.94	.906	.58–1.61	1.63	.084	.94–2.85
Meditation	12.3	8.5	16.7	.66	.329	.29–1.52	1.43	.360	.67–3.07
Avoiding caffeine	8.8	12.3	8.8	1.46	.374	.63–3.36	1.01	.989	.39–2.58
Use alcohol	11.4	21.5	22.5	2.13	.037*	1.05–4.35	2.26	.031*	1.08–4.75
									
Sources of Help									
GP	26.3	21.5	29.4	.77	.382	.43–1.39	1.17	.612	.64–2.12
Counsellor/psychologist	11.4	16.9	17.6	1.58	.222	.76–3.31	1.67	.194	.77–3.60
Family and friends				.92	.757	.55–1.54	1.62	.094	.92–2.86

Categories of Treatments	Mean (SD)	Mean (SD)	Mean (SD)	B	p	95% CI	B	p	95% CI

									
Professional treatments (Scale: 0–6)	1.09 (1.25)	1.06 (1.36)	1.25 (1.49)	-.03	.832	-.34-.27	.15	.374	-.18-.47
Everyday treatments (Scale: 0–4)	2.77 (1.66)	3.13 (1.75)	3.32 (1.50)	.27	.226	-.17-.71	.48	.044*	.01-.94
Complementary treatments (Scale: 0–4)	.54 (.86)	.41 (.76)	.55 (.87)	-.12	.197	-.30-.06	.01	.943	-.19-.20
Dietary treatments (Scale: 0–6)	.45 (.70)	.65 (.87)	.60 (.73)	.16	.064	-.01-.33	.13	.162	-.05-.31

Notes: Professional treatments included; GP, Counsellors and psychologists, anti-depressants, cognitive-behavioural therapy, counselling and reading self-help books. Everyday treatments included; family and friends, exercise, eating chocolate, listening to music, being with pets and doing more things you enjoy. Complimentary treatments included; yoga, massage, relaxation therapy and meditation. Dietary treatments included; cutting out alcohol, taking vitamins, avoiding caffeine and drinking alcohol.

### Associations between actions taken and symptom reduction

Table 3 shows the mean depression scores at post intervention for the users and non-users of each individual treatment. These analyses include individuals from the three intervention conditions combined. Table 3 also lists the results of the linear regressions used to assess the effect of the reported use of each specific treatment, sources of help and treatment categories on depression. Intervention condition and depression at pre-test were controlled for in these analyses. Users of relaxation and those reporting "doing things that they enjoyed' were less depressed. Use of alcohol and the number of professional treatments used were associated with higher levels of depression. These analyses were repeated at six months. There were no major differences in findings at 6 months compared to immediately post-intervention for most of the relationships (Table 4). However, the number of professional treatments used, doing enjoyable things and drinking alcohol, were not longer significant predictors of CES-D scores after six months. Relaxation therapy continued to be associated with lower CES-D scores (B = -.378, p = 0.015). New associations after six months were that CBT was now significantly associated with higher CES-D scores (B = 3.61, p = 0.015), seeing a GP was significantly associated with lower CES-D scores (B = -.2.77, p = .026), and being with pets was associated with lower CES-D scores (B = -3.19, p = .002).

**Table 3:** CES-D scores at post intervention as a function of treatment use and summary of regressions predicting CES-D scores at post intervention

	Treatment Users			Treatment Non Users			Regression Statistics			
Specific Treatments	Mean^b^	SE	N	Mean^b^	SE	N	B	SE	p	95%CI

*Evidence Based*										
Antidepressants	18.46	.86	108	16.90	.50	306	1.58	.98	.106	-.34 – 3.50
CBT	17.99	1.12	62	17.18	.47	352	1.97	1.23	.108	-.44 – 4.38
Self-help books	17.25	.91	96	17.32	.50	318	.24	1.02	.812	-1.77 – 2.25
Exercise	17.30	.55	260	17.31	.71	154	.00	.88	.998	-1.73 – 1.73
Yoga	17.48	1.33	44	17.28	.46	370	.41	1.38	.766	-2.29 – 3.11
Massage	16.19	1.04	72	17.54	.48	342	-1.12	1.12	.997	-3.33 – 1.09
Relaxation therapy	13.38	1.22	51	17.86	.46	356	-3.83	1.29	.003*	-6.35 – -.30
Cut out alcohol	17.68	1.07	68	17.23	.47	346	.48	1.15	.677	-1.78 – 2.73
Use vitamins	16.57	1.17	57	17.42	.47	357	-1.10	1.24	.378	-3.53 – 1.34
										
*Non-evidence based*										
Counselling	19.54	1.28	47	17.02	.46	367	2.17	1.34	.105	-.46 – 4.81
Eating chocolate	16.91	.90	96	17.42	.49	318	-.45	1.01	.659	-2.43 – 1.54
Listening to music	17.61	.59	220	16.95	.63	194	.33	.86	.703	-1.36 – 2.02
Being with pets	16.73	.62	201	17.85	.60	213	-1.35	.85	.114	-3.02 – .32
Doing things you enjoy	15.72	.55	245	19.6	.66	169	-3.95	.84	.000**	-5.60 – -2.31
Meditation	16.42	1.13	61	17.46	.47	353	-.75	1.20	.531	-3.10 – 1.60
Avoiding caffeine	15.88	1.31	45	17.48	.46	369	-1.49	1.36	.273	-4.16 – 1.18
Use alcohol	19.35	.96	85	16.78	.48	329	2.29	1.05	.030*	.22 – 4.37
										
Sources of Help										
GP	18.20	.90	99	17.02	.50	315	1.35	1.01	.183	-.64 – 3.33
Counsellor/psychologist	19.14	1.13	61	16.07	.47	353	2.09	1.20	.083	-.28 – 4.46
Family and friends	17.83	.54	267	16.35	.72	147	1.10	.89	.281	-.65 – 2.85
										
Categories of Treatments										
Professional treatments (0–6)							.74	.32	.021*	.11 – 1.37
Everyday treatments (0–4)							-.39	.26	.133	-.87 – .12
Complementary treatments (0–4)							-.89	.51	.079	-1.88 – .10
Dietary treatments (0–6)							.24	.52	.646	-.78 – 1.23

Notes: a: Linear regression was used for all analyses adjusting for condition and pre-intervention depression.

b: Estimated marginal means adjusted for mean CESD at pre-intervention (21.05).

**Table 4:** CES-D scores at six month follow-up as a function of treatment use and summary of regressions predicting CES-D at six month follow-up

	Treatment Users			Treatment Non Users			Regression Statistics			
Specific Treatments	Mean^b^	SE	N	Mean^b^	SE	N	B	SE	p	95%CI

*Evidence Based*										
Antidepressants	16.65	1.03	93	16.10	.62	253	.50	1.20	.679	-1.86–2.85
CBT	18.62	1.32	55	15.80	.57	291	3.61	1.48	.015*	.71–6.51
Self-help books	15.29	1.10	80	16.54	.60	302	-1.11	1.26	.377	-3.59–1.36
Exercise	16.28	.66	223	16.20	.89	123	.13	1.11	.907	-2.05–2.31
Yoga	15.37	1.61	37	16.35	.56	309	-.82	1.70	.630	-4.17–2.52
Massage	14.54	1.29	58	16.59	.58	288	-1.80	1.42	.206	-4.58-.99
Relaxation therapy	12.59	1.43	46	16.81	.56	300	-3.78	1.55	.015*	-6.82–-.74
Cut out alcohol	15.95	1.30	57	16.31	.58	289	-.27	1.42	.848	-3.07–2.52
Use vitamins	13.91	1.46	46	16.61	.57	300	-2.75	1.56	.079	-5.82-.32
										
*Non-evidence based*										
Counselling	16.22	1.48	44	16.25	.57	302	-.35	1.59	.828	-3.47–2.78
Eating chocolate	15.75	1.07	85	16.41	.61	261	-.71	1.23	.565	-3.13–1.71
Listening to music	16.09	.72	187	16.44	.78	159	-.63	1.07	.554	-2.73–1.47
Being with pets	14.80	.74	172	17.68	.74	174	-3.19	1.05	.002**	-5.25–-1.14
Doing things you enjoy	15.82	.67	211	16.93	.84	135	-1.13	1.07	.292	-3.24-.98
Meditation	15.51	1.39	50	16.23	.57	296	-.26	1.50	.863	-3.21–2.69
Avoiding caffeine	14.03	1.59	38	16.52	.56	308	-2.47	1.67	.140	-5.76-.82
Use alcohol	16.50	1.15	74	16.18	.60	272	.063	1.30	.961	-2.49–2.61
										
Sources of Help										
GP	14.17	1.08	84	16.91	.60	262	-2.77	1.24	.026*	-5.20–-.34
Counsellor/psychologist	15.56	1.37	52	16.32	.57	294	-.68	1.49	.650	-3.60–2.25
Family and friends	16.59	.65	227	15360	.90	119	.81	1.11	.465	-1.37–3.00
Categories of Treatments										
Professional treatments (0–6)							-.15	.39	.707	-.92-.63
Everyday treatments (0–4)							-.44	.33	.179	-1.09-.20
Complementary treatments (0–4)							-1.13	.63	.073	-2.36-.11
Dietary treatments (0–6)							-.85	.61	.181	-2.10-.40

Notes: a: Linear regression was used for all analyses adjusting for condition and pre-intervention depression.

b: Estimated marginal means adjusted for mean CESD at pre-intervention (20.86).

### Severity of depression and its impact on actions taken

**Figure 1:**
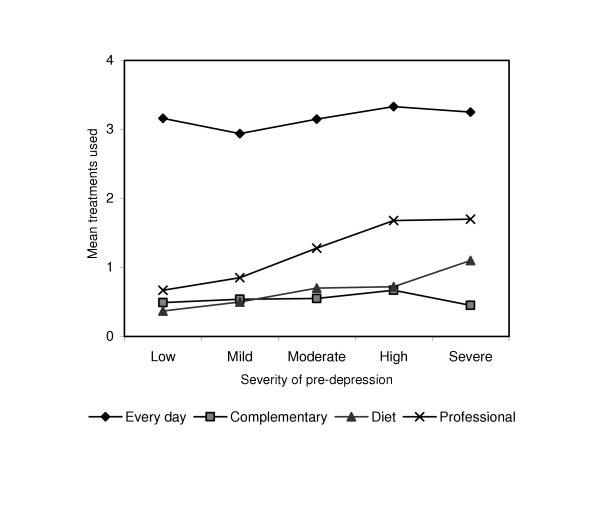
Mean treatments used across severity of depression at pre-intervention.

Table 5 shows the mean depression scores at pre-intervention for the users and non-users of each treatment, including non-evidence based treatments. Table 5 lists the results for the logistic and linear regressions used to assess the effect of depression severity at pre-intervention on the likelihood of using each individual treatment, and on the number of every day, complementary, dietary and professional treatments used. Frequently adopted actions were seeking out family and friends, exercise, listening to music, engaging in pleasant events and being with pets. Taking antidepressants, reading self-help books for depression, using vitamins and using alcohol were associated with higher pretest depression scores. Seeking help from counsellors/psychologists and general practitioners was also associated with higher baseline depression scores, as was the total number of professional treatments. Following Jorm et al. [6] the mean number of treatments used pre-intervention in each category was examined across depression severity (Figure 1). While there was little change in the use of every day, complementary and dietary treatments across severity, the number of professional treatments used increased with increasing severity.

**Table 5:** CES-D scores at pretest as a function of treatment use and summary of regressions predicting treatment use from CES-D at pre-intervention

Specific Treatments^a^	Mean^b^	SE	N	Mean^b^	SE	N	OR	p	95% CI
*Evidence Based*										
Antidepressants	24.08	11.25	108	19.97	9.35	306	1.04	.000**	1.02–1.07
VBT	22.94	10.09	62	20.71	10.00	352	1.02	.108	1.00–1.05
Self-help books	24.44	9.40	96	20.02	10.00	318	1.05	.000**	1.02–1.07
Exercise	21.27	9.83	260	20.66	10.37	154	1.01	.557	.99–1.03
Yoga	22.11	10.15	44	20.92	10.02	370	1.01	.462	.98–1.04
Massage	21.33	9.21	72	20.99	10.21	342	1.00	.808	.98–1.03
Relaxation therapy	21.94	9.84	51	20.92	10.06	363	1.01	.513	.98–1.04
Cut out alcohol	22.72	10.20	68	20.72	9.98	346	1.02	.134	.99–1.05
Use vitamins	24.28	9.57	57	20.53	10.02	357	1.04	.009*	1.01–1.07
									
*Non-evidence based*									
Counselling	23.49	9.72	47	20.73	10.04	367	1.03	.077	1.00–1.06
Eating chocolate	22.68	9.67	96	22.68	10.12	318	1.02	.071	1.00–1.04
Listening to music	21.44	9.81	220	20.60	10.29	194	1.01	.389	.99–1.03
Being with pets	21.78	10.47	201	20.36	9.58	213	1.01	.147	.10–1.03
Doing things you enjoy	21.05	10.04	245	21.04	10.04	169	1.00	.992	.98–1.02
Meditation	20.62	10.44	61	21.12	9.97	353	1.00	.702	.97–1.02
Avoiding caffeine	21.96	9.68	45	20.94	10.08	369	1.01	.522	.98–1.04
Use alcohol	23.85	9.99	85	20.32	9.93	329	1.04	.004*	1.01–1.06
									
Sources of Help									
GP	24.41	9.96	99	19.99	9.83	315	1.05	.000**	1.02–1.07
Counsellor/psychologist	24.11	10.79	61	20.52	9.81	353	1.01	.522	.98–1.04
Family and friends	21.42	10.22	267	20.36	9.67	147	1.01	.289	.99–1.03
Categories of Treatments^c^							B	p	95% CI
							
Professional treatments (0–6)							.03	.000*	.02-.05
Everyday treatments (0–4)							.01	.101	.00-.03
Complementary treatments (0–4)							.00	.640	-.01-.01
Dietary treatments (0–6)							.00	.187	.00-.01

Notes: a: Logistic regressions adjusting for condition.

b: Estimated marginal means adjusted for mean CESD at pre-intervention (21.05).

c: Linear regressions adjusting for condition.

## Discussion

The present study found that use of BluePages was associated with different reports of help seeking behaviours relative to the control condition and MoodGYM. For specific treatment types, compared to MoodGYM, Bluepages use was associated with fewer reports of having taken up CBT, exercise, massage or music. Participants also had lower use of professional treatments, and everyday and complementary categories of treatment. BluePages was associated with decreased use of music, decreased help seeking from friends and family and decreased use of everyday treatments compared to the control condition. Specific self reported actions of participants over the trial period were associated with reduced depression symptoms at endpoint for the use of relaxation, and doing more enjoyable things, but not for other evidenced based and non-evidenced based treatments. Taking antidepressants, reading self-help books for depression, using alcohol and seeking professional treatments were associated with higher baseline depression scores.

### Frequency with which treatments were sought and types of treatments sought

In some respects, these findings are similar to those reported in the recent help seeking literature. First, there is a low level of seeking help from mental health professionals and a preference for everyday treatments, rather than evidence-based treatments. Although figures might be better gauged from representative samples of the population rather than from a sample with high symptoms, only approximately 24% of participants in this study with elevated depression reported seeking the help of a GP. This concords closely with findings by Oliver et al., in the UK that 28% of individuals with high GHQ scores sought help from a general practitioner, and findings in Australia, that 33% of adults with an affective disorder and 56% of those with anxiety disorder did not seek help for their disorder in the last 12 months. A recent study [1] reported that 49% of individuals with a mood disorder perceived the need for care, but only 13% sought help from a mental health professional. Our findings also accord with others that demonstrate that many individuals seek complementary and non-traditional treatments [15,16] rather than treatment through formal mental health services. The most preferred 'treatments' were exercise, doing things you enjoy, listening to music, and being with pets.

### The effect of the specific interventions on reports of help seeking

Jorm et al., [7] demonstrated that the provision of an evidence-based guide led to greater attitudinal change than a short brochure, with those reading the guide more likely to affirm that the endorsed treatments are more likely to be helpful. Those receiving the self help guide were more likely to report that they had tried a self help treatment, and to give advice to someone about it. However, the present study has failed to find strong evidence that the information website BluePages led to the reported initiation of evidence-based treatments either during the intervention or at six months. Compared to the control condition, BluePages participants were no more likely to report having sought any evidence-based specific treatment. Nevertheless, depression improved in the BluePages intervention relative to the control condition at post test [9] and at 6 months. BluePages was associated with a reduction in the use of music, the use of family and friends for support and the use of everyday treatments. We postulated that BluePages might exert its effect through the initiation of self help for evidence based treatments. However, the findings of the study suggest that the help seeking influence of BluePages may be to decrease the use of non-evidence based interventions. For example, seeking support from friends and relatives may be unnecessary, unhelpful, even toxic under certain circumstances and the website may have served to reduce inappropriate help seeking actions. This accords with some adolescent literature that reports that help seeking from younger friends may not be helpful. Moreover, it is possible that BluePages served as a partial substitute for the support and reassurance that might normally be sought from family and friends. The mechanism by which the psychoeducation website exerts it influence on depression symptoms needs further investigation.

The increase in the reported use of CBT in the MoodGYM condition is to be expected since CBT formed the core of the intervention and participants knew CBT was offered in their intervention. The reasons for the increased reporting of massage and exercise are less clear. Neither exercise nor massage was directly encouraged by the MoodGYM site although one of 29 self help exercises mentioned the use of physical activities as a means of rewarding positive thinking. One explanation is that massage and exercise arose a consequence of a reduction in depression symptoms. More generally, this explanation predicts that help seeking is linked to depression improvement across both MoodGYM and BluePages sites. However, although both MoodGYM and BluePages were equally effective in reducing depression symptoms, only MoodGYM was associated with reported increases in these activities.

### Relationships between help sought and a reduction in depression symptoms

The present study found that relaxation therapy, doing enjoyable things, and not using alcohol were associated with lower depression symptoms at post test. These relationships were found controlling for both baseline levels of depression and intervention type. Taking specific evidenced based treatments such as anti-depressant medication (reported by one quarter of the participants) did not predict improvement in depression outcomes. Interpretations of these associations must consider the correlational nature of these relationships as well as the complexity of tracking the uptake of a range of treatments. For example, there may be a number of reasons for failing to find a relationship for anti-depressant medication. First, improvement in mood might be associated itself with the discontinuation of depression medication or the choice not to investigate this option. Other possible explanations are that participants might be unlikely to take antidepressants if they have engaged in online treatment, or the antidepressant effect for those already on antidepressants at commencement of the trial may have stablized at the time of enrolment in the trial. Conversely, the anti-depressant effect may be too small to detect over a six week period if this intervention is taken up later in the course of the trial. Finally, the medication may have been discontinued or used inappropriately and the use of medication might be combined with a number of positive or negative other treatments. These findings however, do also accord with recent data from the UK [3,17] which show that increases in the use of anti-depressant medication for community depression is not associated with improvement in mental health outcomes. A range of methodological problems may be responsible for the absence of these effects, which require tracking longitudinally over a number of data points.

The findings are also interesting in view of the recent NICE guidelines for the management of depression in primary and secondary care which promote the use of guided self help, computerized CBT, and exercise in mild depression, but the use of medication and combined treatments in those with treatment resistant or severe depression [18].

### Depression severity and outcomes

A set of analyses controlling for treatment intervention examined the effect of depression severity on different types of reported help seeking. These findings accord to some degree with those of Jorm et al., [6] who reported that some actions such as taking antidepressants and seeing a doctor became increasingly prevalent with greater severity of symptoms while other actions were less prevalent at higher levels of severity, such as undertaking every day activities, for example, engaging in physical activity.

### Limitations of the findings

There are a number of limitations of the present analysis and design of the study. The major limitation is that the help seeking data are based on self-report. We do not have data on the actual activities undertaken. Secondly, the sample was restricted to that community subgroup of participants who was prepared to participate in a randomised controlled trial of Internet websites. These individuals may have specific preferences for types of treatment not shared by the general community. By virtue of the population from which they were selected, they are likely to be from higher socio-economic groups. Evidence from other work indicates that higher education is associated with the uptake of complementary and alternative treatments [18]. The screening questionnaire was completed by 22.7% of those approached. This level of response is lower than that achieved by US based surveys using the telephone (40%) [16] or surveys with reminders or multiple reminders, or those incorporating participants from established cohort studies. Nevertheless, the rate was consistent with earlier postal surveys of mental health using the electoral roll in Canberra [7]. This lower rate may be due to the lack of personal approach as our broader epidemiological studies of the region yield higher response rates (approximately 60% when telephoned or home visited) [19]. The analyses investigating depression severity and treatments sought are correlational and may reflect differences in the uptake of treatments as a function of changes in depression rather than the influence of particular interventions on the improvement of depression symptoms. The drop out rate was greater in the MoodGYM condition. The increased use of professional, everyday and complementary treatments in the MoodGYM condition may be due to the additive effect of increased use of a number of the individual specific treatments.

Despite these shortcomings the present paper has a number of strengths over previous reports of help seeking in mental health. It is one of the first trials to examine the effect of interventions on both evidence-based and non-evidence based help seeking using adequate comparison groups. The significant findings are that exposure to one internet site results in significantly greater increased help seeking of evidence based treatments (CBT, massage and exercise), and an increase in a range of help seeking. Exposure to BluePages reduces what may be unhelpful responses to mental health problems – seeking support from family and friends. It also confirms severity of depression as a risk factor for help seeking, and reveals the complexity of the relationship between self reported help and treatment outcomes.

## Conclusion

Models of 'pathways to care' are often limited because they fail to account for the large numbers of consumers who do not have access to health care or those who have preferences not to use traditional health services. These models typically consider only the health services that are within the embrace of service providers, excluding self-care and care provided by the not for profit sector and other community agencies. There is clearly the opportunity to pursue broader models of how help seeking may result in better outcomes of those with mental health problems. More needs to be understood about the preferences the community has for various types of treatments (see for example [20,21]) the ways in which people with mental health problems seek both traditional and non-traditional help [10], and the methods which may prove effective in improving the rate and type of help seeking. Further randomised controlled trials are required which examine the effect of various interventions on the actual uptake of help seeking for evidence-based treatments.

## Competing interests

The author(s) declare they have no competing interests.

## Authors' contributions

HC and KG conceived and designed the study. HC drafted the article and KG revised it critically for content. LL undertook the data analyses, drafted method and results sections and contributed comments to the final draft. AM critically revised the content of the paper and its analyses, and LB contributed to intellectual content and data interpretation. All gave final approval of the final manuscript to be published.

## Pre-publication history

The pre-publication history for this paper can be accessed here:


